# Morphometric analysis of the skull base and palatal regions for gender identification using CBCT: a retrospective study

**DOI:** 10.7717/peerj.18127

**Published:** 2024-09-24

**Authors:** Asmaa Uthman, Hesham Marei, Walid Elsayed, Sura F. Al-Bayati, Hawraa Shams Aldeen, Shishir Shetty, Musab Hamed Saeed, Natheer H. Al-Rawi

**Affiliations:** 1 Diagnostic and Surgical Dental Sciences Department, Gulf Medical University, College of Dentistry, Ajman, United Arab Emirates; 2 Department of Oral & Craniofacial Health Sciences, University of Sharjah, College of Dental Medicine, Sharjah, United Arab Emirates; 3 Clinical Sciences Department, Ajman University, College of Dentistry, Ajman, United Arab Emirates

**Keywords:** Cone beam computed tomography, Sexing, Discrminant analysi, Skull base

## Abstract

**Objectives:**

The objectives of this study were to evaluate the accuracy of morphometry of skull base and palate in gender discrimination using cone beam computed tomography (CBCT) scanning and to assess the accuracy of the results among a sample of the Arab population

**Materials & Methods:**

Using CBCT scans, a cross-sectional analysis was conducted on 142 consented patients who underwent various dental procedures at the University Dental Hospital, Sharjah (UDHS). Of these patients, 70 were females and 72 were males, with respective means of 38.5 and 36.2 years. Eleven parameters related to skull base and palatal region were measured on the CBCT scans by two expert radiologists followed by statistical analysis.

**Results:**

There was significant gender-based difference in the mean palatal width (PW) (*p* = 0.001), mean palatal height (PH) (*p* = 0.005). Among other skull base region parameters that were significant in term of gender-based difference like; the clivus length (CL) (*p* < 0.001), occipital condyle height (OCH) (*p* < 0.001), basal angle (BA) (*p* = 0.006) and transverse diameter of foramen magnum (*p* = 0.003). Only palate variables showed a significant age difference. Discriminant analysis related to gender showed that occipital condyle height was the most accurate and best discriminator among the skull base region parameters.

**Conclusion:**

The use of discriminant analysis in CBCT based on skull base and palatal region variables provides an efficient method for determining gender, which is particularly valuable in forensic science and anthropological research.

**Significance of study:**

Accurate gender identification is crucial in forensic investigations, and the skull base region, being a stable and sexually dimorphic anatomical feature, can serve as a reliable marker for this purpose.

## Introduction

Forensic identification is a crucial aspect of forensic science which uses scientific techniques to identify unknown individuals or provide evidence for criminal investigations (Issrani et al., 2022). Gender determination is one area of focus in forensic identification, as it can provide valuable information in the identification process (Nagare et al., 2018).

Cone beam computed tomography (CBCT) imaging has emerged as a powerful and superior tool in the subjective evaluation of the craniofacial complex. This advanced imaging technology provides detailed three-dimensional images of the dental and craniofacial structures, offering significant advantages over traditional two-dimensional radiography methods.

One of the key strengths of CBCT lies in its ability to capture high-resolution images with minimal distortion. Traditional radiographic techniques, such as panoramic and cephalometric imaging, often suffer from overlapping structures, leading to a lack of clarity and precision. In contrast, CBCT produces clear, detailed images that allow for a comprehensive assessment of the entire craniofacial region. The three-dimensional visualization provided by CBCT enables accurate linear measurement of craniofacial lines and angles, aiding in forensic identification (Ajmal et al., 2023; Shaweesh et al., 2023). CBCT provides highly detailed 3D images of the cranium, allowing for precise measurements of various cranial characteristics that can be used to determine gender (Moreira et al., 2009). CBCT offers several advantages over traditional methods such as visual inspection or manual measurements, as it provides more precise and objective measurements and is less susceptible to error and subjective interpretation (Aksoy et al., 2016). Additionally, CBCT can provide information about a person’s ethnicity, age, and ancestry, which can be beneficial for forensic investigations (Zamani Naser & Panahi Boroujeni, 2014). Radiography, including CBCT scans, is commonly used to compare the palatal width and height in forensic identification (Magat & Ozcan, 2019). CBCT can also be used to evaluate abnormalities of the skull base region, which can provide crucial information for forensic investigations (Marques et al., 2012). As a person matures, the shape and size of the skull base region change, and these changes can be observed in CBCT images (Magat & Ozcan, 2019). This information can be useful in cases involving unidentified remains where the age of the person is unknown.

The purpose of this study was to evaluate the accuracy of morphometry of palatal and skull base region in gender discrimination using CBCT scanning and to assess the accuracy of the results among a sample of the Arab population. The research rationale is to provide vital insights into the usefulness and precision of CBCT in forensic science by examining these anatomical landmarks within a specific demography, such as the Arab community. Improving the accuracy of gender identification using CBCT can greatly enhance forensic investigations, helping to solve instances involving unidentified individuals and making valuable contributions to the wider area of forensic anthropology and science.

## Patients & Methods

The radiographic study analysis included 142 CBCT scans of consented patients who underwent different dental operations at the University Dental Hospital, Sharjah (UDHS). Each participant provided written informed consent in accordance with the specific CBCT procedure. This study followed all protocols laid out in the 2014 Declaration of Helsinki and had the approval of the University of Sharjah’s Human Subjects Research Ethics Committee (REC-19-01-24-01). We determined an *a priori* sample size of 140 using the G power analysis software, with the following parameters: effect size = 0.5, error probability = 0.05, and study power = 0.9. The imaging settings used for the scans were as follows: a 17 ×13 cm field of view, 85 kVp, 7 mA, 14 s exposure, and a voxel size of 0.25 mm. The scanner was Galileo’s Comfort CBCT from Sirona Dental Systems in Bensheim, Germany. The CBCT images were displayed on a 23-inch DELL monitor with 1920 ×1080 pixels using the SIDEXIS program. The CBCT pictures were reviewed by two experienced dental radiologists (AU and SS), and a third expert (NA) was brought in to help out if the two of them couldn’t come to a consensus. These differences could be due to poor picture quality, differences in the chosen CBCT slice used to determine distances, or differences in the recognition of anatomical landmarks.

### Inclusion criteria

To ensure accurate measurements of the palate and other craniometric measurements, the study must only use high-quality images with the right field of view (FOV), high spatial resolution, and sufficient image contrast. This will allow for precise identification of the predetermined landmarks. Regarding dentition status, right and left maxillary first molars must be present.

Furthermore, it is imperative that the sample comprises an equal proportion of males and females who were of similar wide age range which is between 11–75 years for both genders, to guarantee a fair and equitable gender representation.

### Exclusion criteria

The study excluded CBCT scans for several reasons, such as previous orthodontic treatment or any dental restorations that obscure the CEJ, medical condition involving abnormalities in the skull base region, osteomyelitis, cysts, or tumors affecting this region. Additionally, scans that did not meet the predetermined measurement standards or produced low-quality images in dynamic CBCT scans were also excluded.

172 CBCT scans were initially included in the study; however, 30 of those scans were subsequently excluded for the reasons stated earlier. The two radiologists (AU and SS) performed inter and intra-rater reliability on the linear measurements, resulting in an inter class coefficient (ICC) of 0.90 which signifies a substantial degree of consistency. Each examiner reevaluated 5% of the total sample 15 days after the initial evaluation to ascertain intra-rater reliability; this yielded an ICC of 0.95. Based on these results, it appears that the measurement technique employed in this research is exceptionally dependable.

### Definition of anatomical landmarks used

#### Skull base parameters

**Figure 1:**
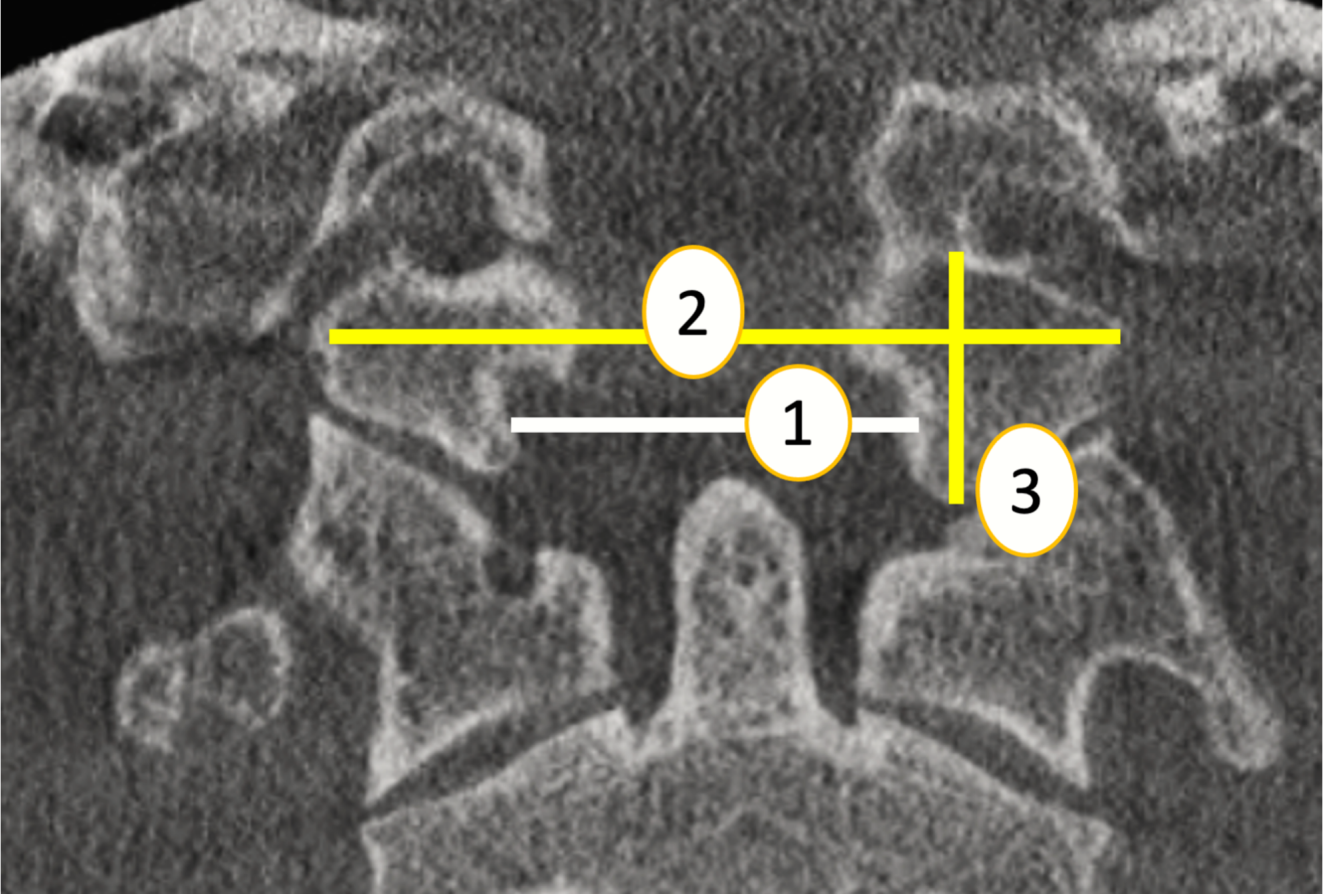
Skull base region parameters (coronal plane)(1:TDFM, 2: WOC , 3: OCH). Source credit: Dr. Asmaa Uthman, Oral Radiologist.

**Transverse diameter of foramen magnum (TDFM**): The maximum horizontal distance of foramen magnum on axial plane. Marques et al. (2012) (Fig. 1, point 1).

**Width of right to left occipital condyle (WOC)**: The greatest distance from the left occipital condyle to the right occipital condyle on axial plane (Abdel-Karim, Housseini & Hashish, 2015). (Fig. 1, point 2).

**The occipital condyle height (**right and left) **(OCH)** was measured on coronal CT images as the length of a line centered in the occipital condyle between the midpoint of its superior articular surface and the midpoint of its inferior articular surface (Abdel-Karim, Housseini & Hashish, 2015) (Fig. 1, point 3).

On a midline sagittal image, **the height of the crista galli (HCG)** and the distance between the crista galli and anterior tuberculum sellae (CG-ATS) were measured.

**Figure 2:**
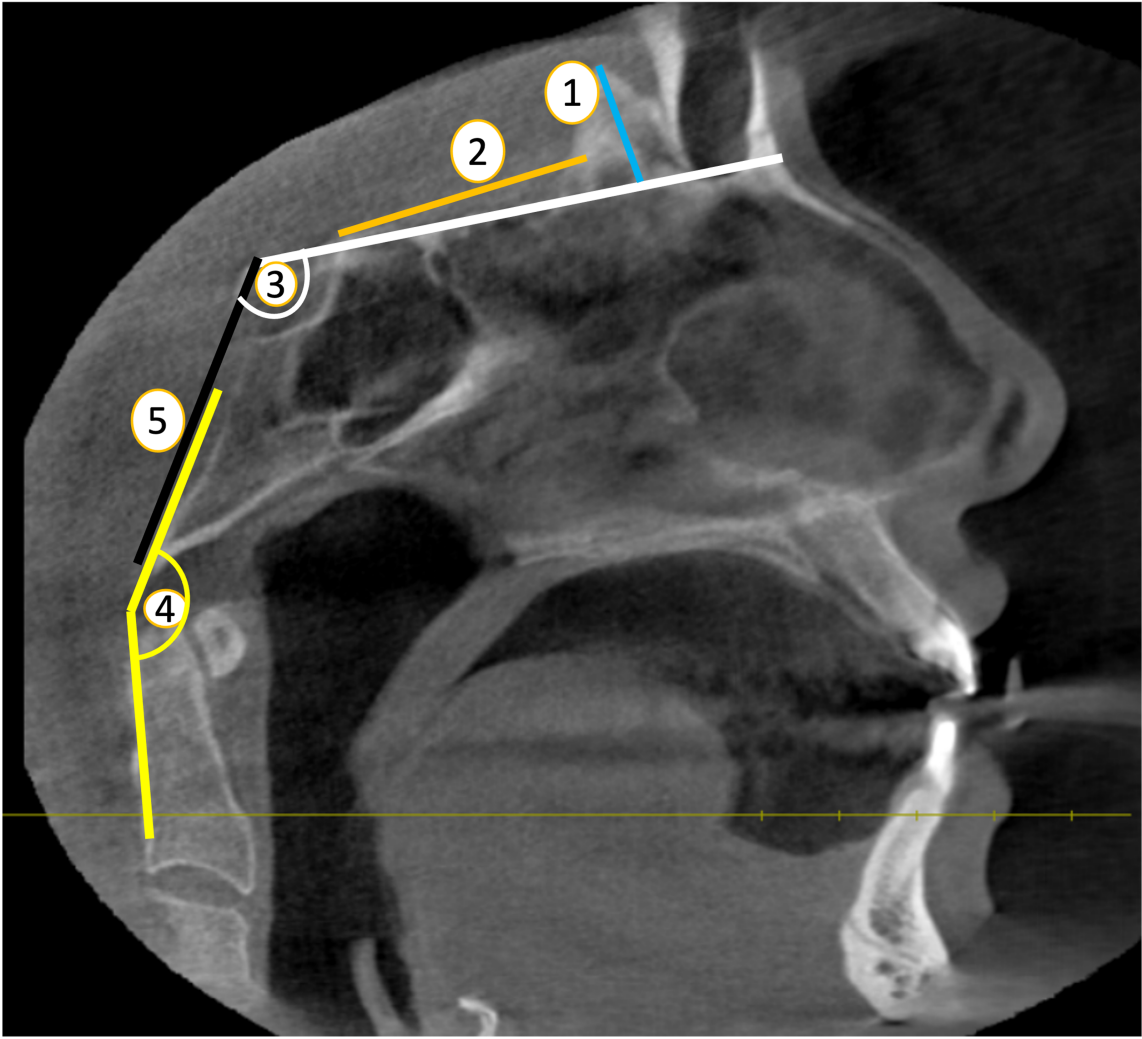
Skull base parameters (Sagittal plane) (1. HCG ; 2.-CG-ATS ; 3. BA; 4. CL; 5. CCA). Source credit: Dr. Asmaa Uthman, Oral Radiologist.

**HCG** was defined as the maximum distance between the apex and base of the crista galli. (Marques et al., 2012) (Fig. 2, point 1).

The **CG-ATS** was determined by measuring the distance from the back edge of the crista galli to the area of the anterior tuberculum sellae, extending to the end of the planum sphenoidale (Batista et al., 2015) (Fig. 2, point 2).

In order to find the **basal angle (BA)**, two lines were drawn on the sagittal plane, one from the nasion to the dorsum sellae and the other from the dorsum sellae to the basion (Botelho & Ferreira, 2013) (Fig. 2, point 3).

**The clivus-canal angle (CCA)** is determined by measuring the angle formed by a line extending from the lower one-third of the clivus and a line extending from the lower back part of the C-2 vertebral body to the upper back portion of the dens on the sagittal plane. (Botelho & Ferreira, 2013) (Fig. 2, point 4).

**Clivus length (CL)** was determined by calculating the distance between the dorsum sellae and the bason on a midline sagittal plane (Dufton et al., 2011) (Fig. 2, point 5).

**Figure 3:**
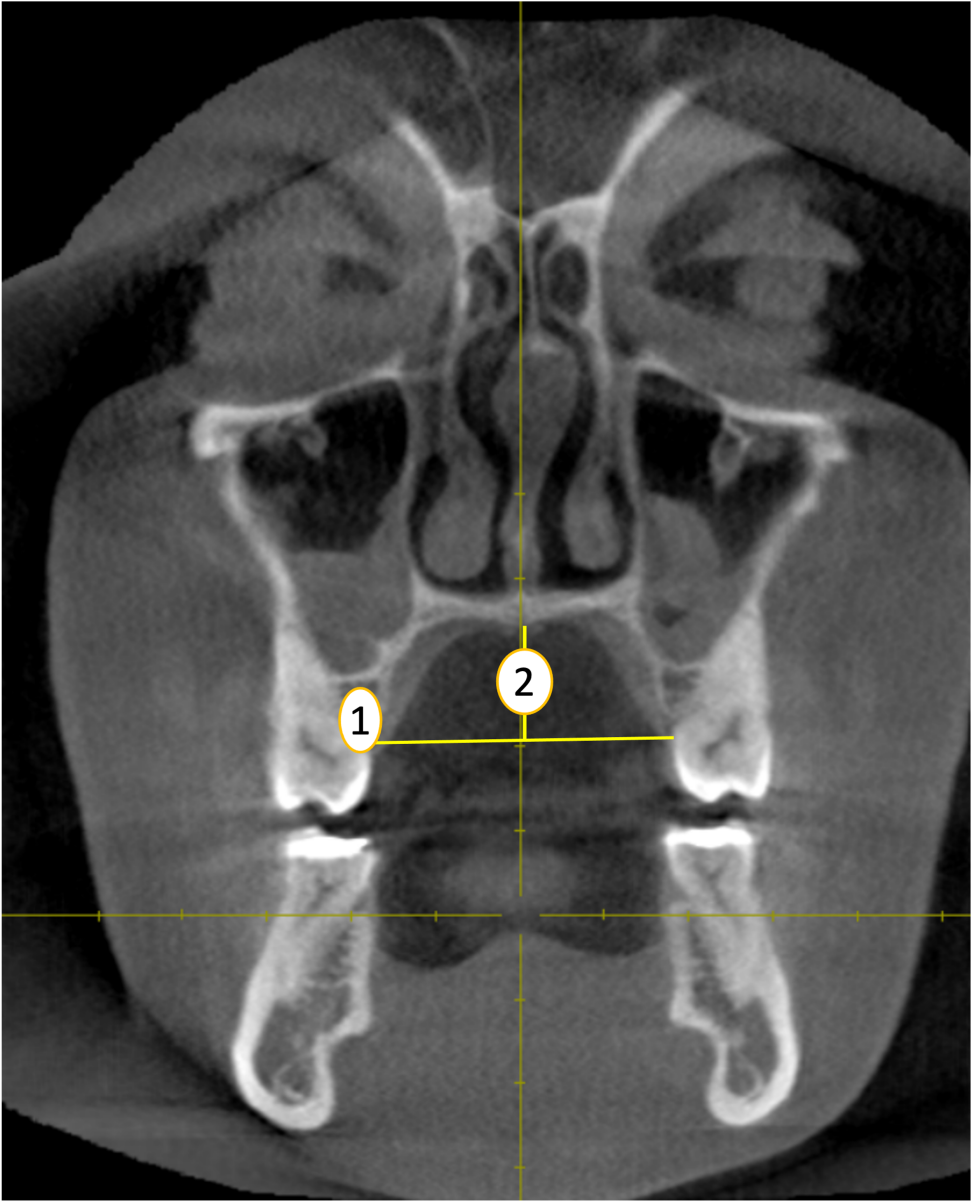
Palatal width (1) and height (2) measurements (coronal plane). Source credit: Dr. Asmaa Uthman, Oral Radiologist.

**Palatal width (PW):** The distance between the cemento-enamel junction of the left and right first molars from coronal plane (Spradley & Jantz, 2011) (Fig. 3, point 1).

**Palatal height (PH):** The distance of the perpendicular line drawn from midpalatal region intersecting the horizontal line connecting the cemento-enamel junction of the left and right first molars from coronal plane. (Spradley & Jantz, 2011) (Fig. 3, point 2).

### Statistical analyses

All quantitative variables were presented as mean ±SD. The difference between females and males was tested on different measured variables using independent sample *t*-test to demonstrate significant difference, and the difference between age categories was tested using one-way ANOVA. A significant difference is recognized as *P* < 0.05. Pearson’s correlation was used to examine all variables.

A multi-regression analysis called discriminant analysis was used to predict gender based CBCT values. Each craniometric variable was analyzed using simple discriminant analysis. Significant discriminant parameters then entered several discriminant processes. Discriminant analysis yielded these parameters:

1. Discriminant regression model equation: predicts gender-based discrimination power: D = ±constant value ±vX1 ±v2X2 ±v3X3 ±viXi, where D is the discriminant score, v is the unstandardized coefficient, and X is the studied variable.

2. *P* value (discriminant regression model): determine statistical significance of independent variables (predictors) at *P* < 0.05.

Discriminant score (D): the equation was used to calculate this value from the predictor variables. Its value is compared to D (model-provided and ROC curve cut off value). If greater than the reference figure, a male gender is assumed; if smaller, a female gender.

Correctly predicted group membership percentage: crucial to proving the model’s gender prediction accuracy. The formula is: (number of successfully predicted subjects / gender type / total subjects / gender) ×100%. It also uses sensitivity and specificity to detect reference D score (cut off value) from the ROC curve.

Functions at group centroid: when the calculation uses the mean value of a given CBCT measurement for a gender. It shows how widely apart each gender’s calculated D is.

After excluding non-significant factors, discriminant analysis was performed on all significant predictors in one model.

## Results

### Gender differences among age

There was no significant difference between females and males regarding age, achieving the base line characteristics between the two genders as shown in Table 1.

**Table 1:** Gender differences among age.

**Variable**	**Males (*n* = 72)**		**Females (*n* = 70)**		***t* value**	***P* value**
	**Mean ± SD**	**Range** ** (year, month)**	**Mean ±SD**	**Range** ** (year, month)**		
**Age**	38.51 ± 17.34	11, 11–75.2	36.20 ± 16.75	11, 9–75, 4	0.808	0.420

### Gender differences among measured variables

There was no significant difference between females and males regarding CCA and WOC; however, females showed significantly high BA (120.34° ± 5.21) as compared to males (118.08° ± 4.41) as shown in Table 2.

**Table 2:** Gender differences among measured variables.

**Variable**	**Males (*N* = 72)**	**Females (*n* = 70)**	***t* value**	***P* value**
	**Mean ± SD**	**Mean ± SD**		
**HCG** ** (mm)**	10.90 ± 2.63	11.10 ± 2.28	0.475	0.636
**CG-ATS** ** (mm)**	34.42 ± 3.54	32.74 ± 2.80	3.137	0.002^*^
**BA** ** (** ^∘^ **)**	118.08 ± 4.41°	120.34 ± 5.21°	2.791	0.006^*^
**CL** ** (mm)**	44.79 ± 3.17	42.53 ± 2.94	4.386	<0.001^*^
**OCHR** ** (mm)**	11.07 ± 1.58	9.84 ± 1.32	5.032	<0.001^*^
**OCHL** ** (mm)**	11.06 ± 1.58	9.83 ± 1.32	5.019	<0.001^*^
**CCA** ** (** ^∘^ **)**	162.05 ± 8.23	160.74 ± 9.43	0.876	0.383
**WOC** ** (mm)**	23.73 ± 2.49	24.43 ± 2.37	1.718	0.088
**TDFM** ** (mm)**	30.19 ± 2.51	28.87 ± 2.70	2.997	0.003^*^
**PH** ** (mm)**	14.89 ± 3.79	13.26 ± 2.90	2.855	0.005^*^
**PW** ** (mm)**	37.08 ± 3.73	35.04 ± 3.37	3.407	0.001^*^

**Notes.**

HCG height of the crista galli CG-ATS Crista galli-anterior tuberculum sellae BA Basal angle CL Clivus length OCHR Occip condylar height R OCHL Occip condyar height L CCA Clivus canal angle WOC Width of right and left occip condyle TDFM Transverse diameter of foramen magnum PH Palatal height PW Palatal width

* Significantly different at *p* < 0.05. Statistical analysis was carried out using independent sample *t* test to elucidate significant difference between females and males.

The males showed significantly high BA (118.08 ^∘^ ± 4.41), CG-ATS (34.42 mm ± 3.54), CL (44.79 mm ± 3.17), OCHR (11.07 mm ± 1.58), OCHL (11.06 mm ± 1.58), TDFM (30.19 mm ± 2.51), PH (14.89 mm ± 3.79), and PW (37.08 mm ± 3.73) as compared to females (120.34° ± 5.21, 32.74 mm ±2.80, 42.53 ±2.94, 9.84 mm ±1.32, 9.83 mm ± 1.32, 28.87 mm ± 2.70, 13.26 mm ± 2.90, and 35.04 mm ± 3.37 respectively) as shown in Table 3.

**Table 3:** Age differences among measured variables.

**Variable**		**Age groups**						**F Value**	***P* value**
		**Gp 1 (<20 years) (*n* = 24)**	**GP 2 (20–29 years) (*n* = 30)**	**GP 3 (30–39 years) (*n* = 32)**	**GP 4 (40–49 years) (*n* = 16)**	**GP 5 (50–59 years) (*n* = 22)**	**GP 6 (>60 years) (*n* = 18)**		
**HCG** ** (mm)**	**Mean ± SD**	10.58 ± 2.79	11.17 ± 2.21	11.28 ± 2.26	11.46 ± 2.99	10.47 ± 2.56	11.02 ± 2.24	0.562	0.729
	**Range**	4–15	8–15	8–16	7–16	6–17	7–16		
**CG-ATS** ** (mm)**	**Mean ± SD**	33.92 ± 3.71	33.58 ± 3.16	34.40 ± 3.91	34.19 ± 2.15	32.30 ± 3.02	32.80 ± 2.61	1.449	0.211
	**Range**	28–45	28–40	27–49	31–40	28–37	29–39		
**BA** **(** ^∘^ **)**	**Mean ± SD**	117.55 ± 4.84	119.62 ± 4.96	120.99 ± 4.59	117.88 ± 5.22	119.30 ± 4.86	118.51 ± 4.94	1.767	0.124
	**Range**	110.90–130.20	109.90–132.60	111.90–133.80	110.50–126.00	107.70–128.70	111.40–128.00		
**CL** ** (mm)**	**Mean ± SD**	43.84 ± 3.07	43.57 ± 2.89	43.85 ± 2.99	44.17 ± 3.20	43.62 ± 3.86	42.94 ± 3.98	0.288	0.919
	**Range**	39–48	39–51	39–49	37–50	36–50	34–50		
**OCHR** ** (mm)**	**Mean ± SD**	10.44 ± 1.51	10.12 ± 1.45	10.43 ± 1.85	10.52 ± 1.46	10.69 ± 1.42	10.79 ± 1.71	0.537	0.748
	**Range**	8–14	7–14	8–16	8–13	8–13	8–13		
**OCHL** ** (mm)**	**Mean ± SD**	10.44 ± 1.54	10.12 ± 1.45	10.42 ± 1.86	10.51 ± 1.46	10.70 ± 1.43	10.78 ± 1.71	0.531	0.752
	**Range**	8–14	7–14	8–16	8–13	8–13	8–13		
**CCA** **(** ^∘^ **)**	**Mean ± SD**	159.68 ± 9.09	161.15 ± 9.63	158.48 ± 7.12	163.74 ± 9.08	165.43 ± 7.53	162.29 ± 9.68	2.128	0.066
	**Range**	141.70–175.50	137.50–178.30	142.40–172.90	145.00–176.90	152.40–176.20	146.10–175.00		
**WOC** ** (mm)**	**Mean ± SD**	24.78 ± 2.18	24.14 ± 2.35	24.16 ± 2.78	24.06 ± 2.75	23.74 ± 1.86	23.27 ± 2.72	0.881	0.496
	**Range**	20–29	20–30	20–30	19–30	19–27	19–30		
**TDFM** ** (mm)**	**Mean ± SD**	28.72 ± 2.82	29.84 ± 1.87	29.89 ± 3.04	29.68 ± 2.42	29.97 ± 3.03	28.85 ± 2.72	0.994	0.424
	**Range**	21–33	25–34	24–37	25–34	25–37	25–35		
**PH** ** (mm)**	**Mean ± SD**	13.65 ± 3.07^a^	15.20 ± 3.08^b^	14.76 ± 2.65^b^	15.64 ± 4.59^b^	13.25 ± 3.72^a^	11.20 ± 2.56^ab^	4.952	<0.001^*^
	**Range**	8–22	6–21	9–19	7–22	7–21	8–16		
**PW** ** (mm)**	**Mean ± SD**	34.02 ± 3.38^a^	34.28 ± 3.34^a^	36.23 ± 3.57^ab^	38.30 ± 3.08^b^	36.87 ± 2.97^ab^	38.66 ± 3.47^b^	7.441	<0.001^*^
	**Range**	27–41	30–42	29–45	34–44	31–43	34–45		

**Notes.**

HCG height of the crista galli CG-ATS Crista galli-anterior tuberculum sellae BA Basal angle CL Clivus length OCHR Occip condylar height R OCHL Occip condyar height L CCA Clivus canal angle WOC Width of right and left occip condyle TDFM Transverse diameter of foramen magnum PH Palatal height PW Palatal width

* Significantly different at *p* < 0.05. Statistical analysis was carried out using one way ANOVA test followed by Tukey’s pairwise comparisons test to elucidate significant difference different age groups. Groups with different letters are significantly different from each other.

### Age differences among variables

There was no significant difference between all age groups regarding all skull base parameters, however PH and PW showed significant age changes. Regarding PH, more than 50 year showed significantly lower PH as compared to age groups (20–29), (30–39), and (40–49) y, while there was no significant difference between other age groups. Regarding PW, less than 20 year and age group (20–29) year showed significant lower PW as compared to age groups (30–39) and (40–49), and more than 50 year, while there was no significant difference between other age groups, Table 3.

### Correlation between different studied craniometric variables in female and male groups

The results of correlation were shown in Table 4 for males and females. Regarding males, there was a significant positive correlation for CL with OCHR/OCHL, CCA with PH, OCHR with OCHL. Most of significant correlations were weak to moderate except for OCHR with OCHL that showed a completely strong positive correlation (*r* = 1.00, *P* < 0.0). All other studied parameters showed no significant correlation with each other.

**Table 4:** Correlation between different non odontoid studied variables in male and female group.

**Males**	**HCG**	**CG-ATC**	**BA**	**CL**	**OCHR**	**OCHL**	**CCA**	**WOC**	**TDFM**	**PH**	**PW**	**Female**
**HCG**	1	−0.052	−0.050	−0.074	−0.001	0.010	0.023	0.043	−0.025	−0.022	0.058	
**CG-ATS**	0.020	1	−0.049	0.209	0.021	0.029	−0.216	0.013	0.002	0.046	0.068	
**BA**	−0.171	0.036	1	0.026	−0.178	−0.169	−0.530^**^	0.054	0.180	0.070	−0.147	
**CL**	−0.225	0.218	−0.176	1	.269^*^	.273^*^	−0.110	0.109	0.163	0.201	0.014	
**OCHR**	−0.023	0.090	−0.001	0.234^*^	1	1.000^**^	0.091	0.047	−0.167	0.213	0.083	
**OCHL**	−0.023	0.087	−0.002	0.234^*^	1.000^**^	1	0.082	0.043	−0.165	0.212	0.084	
**CCA**	0.057	−0.059	−0.491^**^	0.086	0.043	0.045	1	−0.213	−0.138	−0.125	0.030	
**WOC**	0.060	0.005	−0.037	−0.129	0.051	0.050	−0.052	1	0.335^**^	0.222	0.072	
**TDFM**	−0.128	−0.042	0.114	0.112	−0.043	−0.045	−0.009	0.226	1	0.121	−0.001	
**PH**	−0.114	0.147	−0.084	0.159	−0.101	−0.102	0.251^*^	0.051	0.128	1	−0.160	
**PW**	−0.091	0.048	0.051	0.122	0.181	0.180	0.039	−0.239^*^	0.129	−0.097	1	

**Notes.**

HCG height of the crista galli CG-ATS Crista galli-anterior tuberculum sellae BA Basal angle CL Clivus length OCHR Occip condylar height R OCHL Occip condyar height L CCA Clivus canal angle WOC Width of right and left occip condyle TDFM Transverse diameter of foramen magnum PH Palatal height PW Palatal width

The value in each cell represents the correlation coefficient (r).

** correlation is significant at *p* < 0.05, 0.01.

Regarding females, there was a significant positive correlation for CL with OCHR/OCHL, WOC with TDFM. Most of significant correlations were weak to moderate except for OCHR with OCHL that showed a completely strong positive correlation (*r* = 1.00, *P* < 0.0). All other parameters showed no significant correlation with each other.

### Discriminant analysis using studied variables to discriminate between genders

Substituting the researched variable (s) values into the discriminating model equation to calculate D will assist predict gender. Comparing the D value to a reference value (ROC curve following model equation prediction D score). Calculated D larger than reference D indicates male gender, whereas less than reference D indicates female gender. The model calculated for the studied parameters was statistically significant regarding CL, OCHR, OCHL, PH and PW. Among the significant CBCT measurements included, OCHR/OCHL was the most accurate and best discriminator, followed by CL, while BA was the least accurate one (Table 5).

**Table 5:** Discriminant analysis using studied variables to discriminate between genders.

**Clivus length**				
D = −14.278 + 0.327 CL; Wilk’s Lambda = 0.879, *P* < 0.001 (S)				
**Percentage of accurately predicted group membership**	**Male**	**Female**	**Overall**	**AUC**
	65.3%	72.9%	69.0%	0.705
**Functions at group centroids**	0.363	−0.373	Classified male if D >−0.874	
**Occipital condyar height R**				
D = −7.182 + 0.687 OCHR; Wilk’s Lambda = 0.847, *P* < 0.001 (S)				
**Percentage of accurately predicted group membership**	**Male**	**Female**	**Overall**	**AUC**
	66.7%	65.7%	66.2%	0.722
**Functions at group centroids**	0.416	−0.428	Classified male if D >−0.577	
**Occipital condyar height L**				
D = −7.153 + 0.684 OCHL; Wilk’s Lambda = 0.847, *P* < 0.001 (S)				
**Percentage of accurately predicted group membership**	**Male**	**Female**	**Overall**	**AUC**
	66.7%	67.1%	63.4%	0.720
**Functions at group centroids**	0.414	−0.432	Classified male if D >−0.537	
**CG-ATC**				
D = −10.497+0.312 CG-ATC, Wilk’s Lambda = 0.934, *P* = 0.002 (S)				
**Percentage of accurately predicted group membership**	**Male**	**Female**	**Overall**	**AUC**
	59.7%	62.9%	61.3%	0.643
**Functions at group centroids**	0.260	−0.267	Classified male if D >−0.833	
**Clivus canal angle**				
D = −18.267 + 0.113 CCA; Wilk’s Lambda = 0.994, *P* = 0.383(NS)				
**Percentage of accurately predicted group membership**	**Male**	**Female**	**Overall**	**AUC**
	47.2%	50.0%	48.6%	NS
**Functions at group centroids**	0.072	−0.076	NS	
**Basal angle**				
D = −24.731+0.207 BA, Wilk’s Lambda = 0.947, *P* = 0.006 (S)				
**Percentage of accurately predicted group membership**	**Male**	**Female**	**Overall**	**AUC**
	55.6%	62.9%	59.2%	0.363
**Functions at group centroids**	0.231	−0.238	Classified male if D >−1.351	
**Width of right and left occip condyle**				
D = −9.895 + 0.411 WOC; Wilk’s Lambda = 0.979, *P* = 0.088 (NS)				
**Percentage of accurately predicted group membership**	**Male**	**Female**	**Overall**	**AUC**
	59.7%	52.9%	56.3%	NS
**Functions at group centroids**	0.142	−0.146	NS	
**Transverse diameter of foramen magnum**				
D = −11.332 + 0.384 TDFM; Wilk’s Lambda = 0.940, *P* = 0.003(S)				
**Percentage of accurately predicted group membership**	**Male**	**Female**	**Overall**	**AUC**
	61.1%	57.1%	59.2%	0.633
**Functions at group centroids**	0.248	−0.255	Classified male if D >−1.822	
**Palate height**				
D = −4.170 + 0.296 PH; Wilk’s Lambda = 0.945, *P* = 0.005 (S)				
**Percentage of accurately predicted group membership**	**Male**	**Female**	**Overall**	**AUC**
	59.7%	60.0%	59.9%	0.632
**Functions at group centroids**	0.239	−0.242	Classified male if D > −0.456	
**Palate width**				
D = −10.131 + 0.281 PW; Wilk’s Lambda = 0.923, *P* = 0.001 (S)				
**Percentage of accurately predicted group membership**	**Male**	**Female**	**Overall**	**AUC**
	59.7%	60.0%	59.9%	0.655
**Functions at group centroids**	0.281	−0.293	Classified male if D > −2.118	

**Notes.**

NS non significant discrimination S significant discrimination at *P* < 0.05

Area under the curve for predicted D score from the model (AUC) was obtained from ROC curve to detect refercnce D score (cut off value).

The highest overall classification accuracy for gender was achieved by incorporating all substantially correlated variables into the regression model (Wilk’s lambda = 0.687, Overall accuracy = 0.779). Following palatal width, this combination also demonstrated that OCH on both the right and left sides was the most effective discriminator.

As a result, the following equation was derived to compute D:

−9.340+0.100PW+0.084PH+0.127TDFM −0.062BA +0.356OCHR+0.100CL, Table 6.

**Table 6:** Discriminant analysis using significant studied variables after exclusion the nonsignificant ones to discriminate between genders.

	**Standardized coefficients**				
**Clivus length**	0.250				
**Occipital condyle height right**	0.516				
**Basal angle**	−0.236				
**CG-ATC**	0.240				
**Transverse diameter of foramen magnum**	0.332				
**Palate height**	0.256				
**Palate width**	0.331				
D = −9.340+0.100PW+0.084PH+0.127TDFM -0.062BA +0.356OCHR+0.100CL Wilk’s Lambda = 0.687, *P* < 0.001 (S)					
**Percentage of accurately predicted group membership**	**Male**	**Female**	**Overall**	**AUC**
	76.1%	79.7%	77.9%	0.859
**Functions at group centroids**	0.660	−0.679	Classified male if D >−0.509	

**Notes.**

BA Basal angle CL Clivus length OCHL Occip condyar height L OCHR Occip condylar height R PH Palatal height PW Palatal width TDFM Transverse diameter of foramen magnum NS non-significant discrimination S significant discrimination at *P* < 0.05

# Multiregreesion test can tolerate OCHR or OCHL not both of them due to their similarity.

Area under the curve for predicted D score from the model (AUC) was obtained from ROC curve to detect refercnce D score (cut off value).

## Discussion

The skull base is crucial in forensic identification due to its unique anatomical features that aid in determining various aspects of an individual’s identity. Gender identification is facilitated by sexual dimorphism in the skull base, with studies showing that discriminant analysis of skull base dimensions can accurately determine an individual’s sex (Spradley & Jantz, 2011; Walker, 2008). This underscores the skull base’s importance in gender identification within forensic anthropology. The present study support this notion were the clivus length, occipital condyle height, basal angle and transverse diameter of foramen magnum were among the other cranium base region parameters that were significantly greater in males than in females. Additionally, the skull base is essential for establishing the biological profile of an individual, including factors such as age, sex, ancestry, and stature. Combined with other skeletal elements, the skull base provides valuable information for comprehensive individual identification (Baryah, Krishan & Kanchan, 2019). Moreover, the skull base is vital for facial reconstruction, serving as the foundation for reconstructing soft tissue features and aiding in visual representation for identification purposes (Bonda, 2018). Furthermore, the skull base is utilized in ancestry estimation, where its features are assessed to determine an individual’s ancestral background. This information is crucial in narrowing down potential matches in missing persons cases or unidentified remains (Pengyue et al., 2021). Advanced techniques like deep neural networks can be employed to analyze the skull base’s morphological characteristics for insights into an individual’s ancestry (Pengyue et al., 2021). Palatal width and height were assessed by Ajanovic et al. (2022), who used the method of geometric morphometry and found that the determination of sex based on the size and shape of the hard palate was possible with an accuracy of 58.57% for women and 66.91% for men in the sample from the population of Bosnia and Herzegovina. The accuracy of their study was slightly lower than that observed in the present study (60%) for women and moderately higher than that of our study (59.7%) for men. In the study conducted by Uthman, Al-Rawi & Al-Timimi (2012), the dimensions of the foramen magnum were examined to determine differences between genders. The researchers found that the circumference and area of the foramen magnum were the most effective parameters for studying sexual dimorphism, with an accuracy of 67% and 69.3% respectively. These findings are similar to the results of the current study, which showed an accuracy of 61.1% for men and 57.1% for women. Through the application of multivariate analysis, it was determined that 90.7% of the FM dimensions of males and 73.3% of the FM dimensions of females were accurately classified by sex (Uthman, Al-Rawi & Al-Timimi, 2012). The height of the occipital condyle has been specifically studied in relation to gender identification in relation to morphological analysis of the foramen magnum (Chethan et al., 2012). They found that the shape of the foramen magnum varied among individuals, and this information could be useful for anthropologists, morphologists, and clinical anatomists. Abo El-Atta et al. (2020) examined the sexual dimorphism of the occipital condyles and foramen magnum in an Egyptian population. Males and females differed significantly in ten dimensions of the occipital condyles and foramen magnum (Abo El-Atta et al., 2020).

The dimensions and characteristics of various anatomical structures, such as the frontal sinuses, maxillary sinuses, and mastoid process, have been analyzed using discriminant analysis to determine their potential for gender determination. Discriminant analysis methods have proven to be effective in accurately categorizing individuals based on their gender. Jayakrishnan, Reddy & Kumar (2023) conducted a study using discriminant function analysis and found that 91.3% of females and 50% of males correctly identified their gender based on paranasal sinus dimensions. These findings demonstrate the potential of discriminant analysis methods in CBCT for gender identification. Recently researchers have also found success in gender determination using parameters like intra mastoid diameter and inter zygomatic width (Jayakrishnan, Reddy & Kumar, 2023; Albalawi et al., 2019). Some of the recent studies have also investigated the CBCT variables in the mandible for possible role in gender determination (Rathithya et al., 2023). Rathithya et al. (2023) found that the distance between the anterior and posterior mandibular foramen is good predictor for gender determination. Another CBCT based study by Albalawi et al. (2019) reported that angle formed by the intersection of lines from the left and right gonion to mention could be useful in gender determination. However, another recent study by Bakan et al. (2022) revealed that CBCT based gonial angle measurements were not very precise in determining gender.

The future scope of work in this field would depend on the application of machine learning algorithms. Recent studies have revealed that the Gaussian naive Bayes machine learning model showed high accuracy (90%) in gender determination using CBCT (Baban & Mohammad, 2023).

### Strength of the study

The use of discriminant analysis to assess the accuracy of gender discrimination based on morphometric variables is a notable strength. This statistical technique helps in identifying which variables (such as occipital condyle height) are most effective in distinguishing between genders.

### Limitations of the study

The ethnic diversity among the participants in this study could potentially impact the precision of the measurements taken, thus constituting a potential limitation. For future investigations to be more precise, a larger sample size and the categorization of Arabs by ancestry will be required.

## Conclusion

The use of discriminant analysis in CBCT for gender identification offers several advantages. It provides an efficient method for determining gender, which is particularly valuable in forensic science and anthropological research. Discriminant analysis related to gender showed that occipital condyle height to be the most accurate and best discriminator among the skull base region parameters (AUC = 0.722). The overall accuracy for gender discrimination using all parameter was 77.9%. Palatal dimensions yielded accuracy in females’ identification by 60%. By analyzing the dimensions and characteristics of specific anatomical structures, discriminant analysis can provide reliable and accurate results for gender determination.

##  Supplemental Information

10.7717/peerj.18127/supp-1 Supplemental Information 1 Raw CBCT data

## Additional Information and Declarations

### Data Availability

The following information was supplied regarding data availability: The raw data is available in the Supplemental File.
